# The impact of gut microbiome on intrahepatic cholestasis of pregnancy—systematic literature review

**DOI:** 10.3389/fmed.2026.1859397

**Published:** 2026-06-17

**Authors:** Florence Julie Donzé, Nadine Eli, Nicoletta Di Simone, Barbara Baur Cavegn, Martin Mueller

**Affiliations:** 1 Faculty of Medicine, University of Bern, Bern, Switzerland; 2 Department of Biomedical Sciences, Humanitas University, Milan, Italy; 3 IRCCS Humanitas Research Hospital, Milan, Italy; 4 Department of Obstetrics, Lindenhofgruppe AG, Bern, Switzerland; 5 Institute of Biochemistry and Molecular Medicine, University of Bern, Bern, Switzerland

**Keywords:** bile acid metabolism, dysbiosis, gut microbiome, ICP, intrahepatic cholestasis of pregnancy, pregnancy complications, pregnancy-specific liver disease

## Abstract

In recent years, there has been a growing interest in the gut microbiome and its potential role in the etiopathogenesis of both gastrointestinal and extraintestinal diseases. Dysbiosis, characterized by a pathological alteration in the composition of the gut microbiome, has been implicated in various gastrointestinal diseases. This paradigm extends to pregnancy-specific conditions, including intrahepatic cholestasis of pregnancy (ICP). ICP exhibits a multifactorial etiopathogenesis, involving hormonal, genetic and environmental factors, among others. Despite growing scientific evidence, there is currently a lack of comprehensive reviews that specifically examine the causal mechanisms through which gut microbiota dysbiosis might contribute to the pathogenesis of ICP, as well as the resulting implications for the development of new targeted therapeutic approaches. Notably, shifts in microbial taxa and the depletion of bacteria involved in certain metabolic pathways have been observed in ICP. These findings suggest that alterations in the gut microbiome composition may contribute to the pathophysiology of ICP. Such microbiome-associated alterations may have important implications for risk stratification and early identification of patients at increased risk of adverse maternal and fetal outcomes. Further investigation into these microbial changes and molecular pathways could offer novel insights and identify potential pharmacological targets for ICP development and management. In particular, modulation of the gut microbiome could represent a future adjunctive strategy to existing therapeutic approaches, potentially improving disease monitoring and individualized management. The precise role of gut microbiome composition in the management and treatment of ICP is still not fully understood, highlighting the need for a systematic review to synthesize existing evidence and identify critical gaps relevant to the future development of screening, prevention, and targeted therapeutic strategies.

## 1 Intrahepatic cholestasis of pregnancy

Intrahepatic cholestasis of pregnancy (ICP) is both a primary liver and pregnancy-specific disease (1). It develops predominantly in the second or third trimester of pregnancy and always resolves after delivery (2). The pathomechanism of ICP is complex and not fully understood but recent studies point towards the association between the gut microbiome and pregnancy-related diseases (1, 3).

### 1.1 Incidence

**Figure 1:**
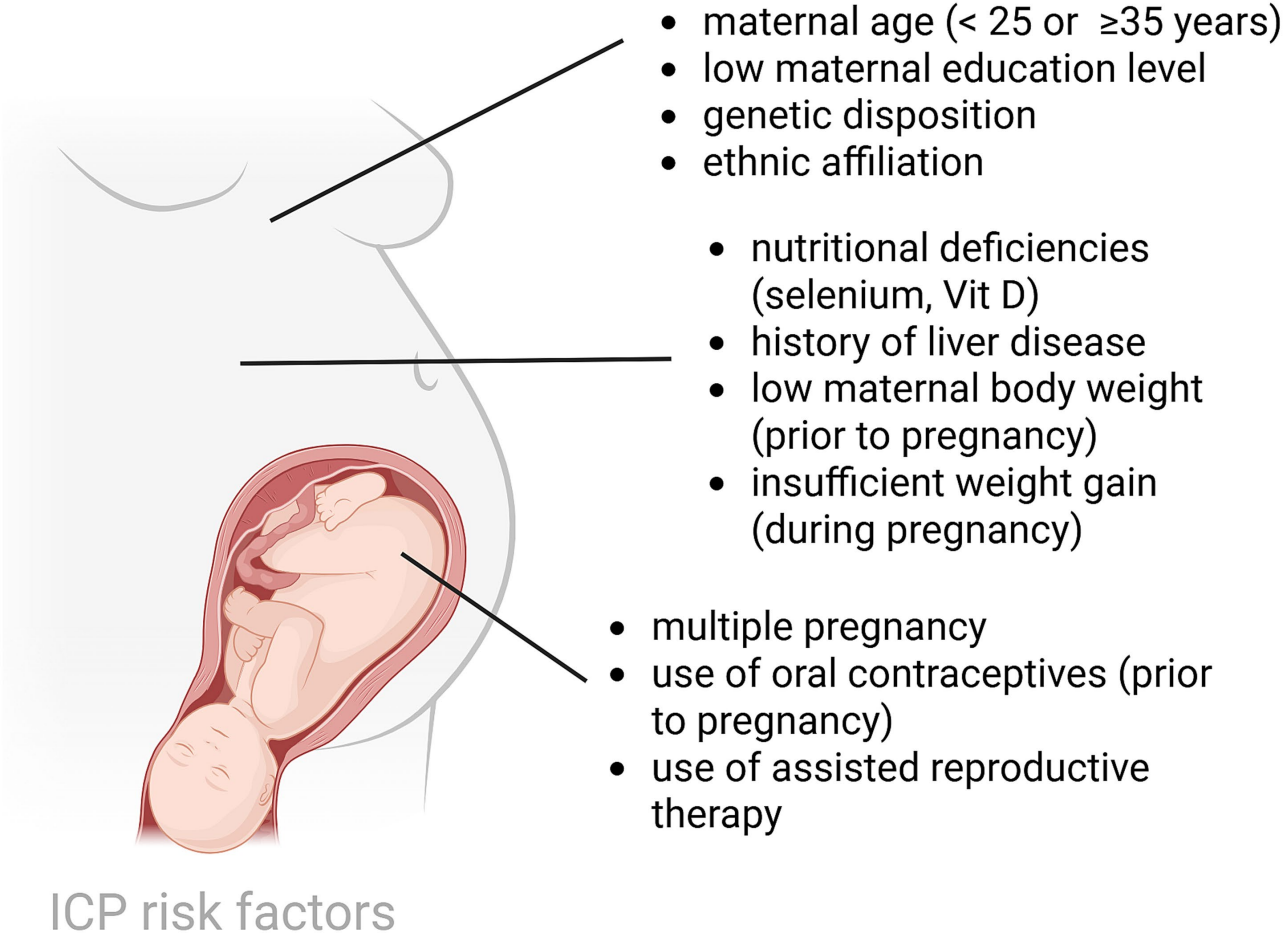
ICP risk factors. Several risk factors may predispose a pregnant individual to develop intrahepatic cholestasis of pregnancy (ICP), including genetic, demographic, nutritional, and hormonal influences.

The incidence of ICP varies among different ethnic groups as well as geographically with incidence ranging between 0.3–27.6% (1, 4). In the USA the incidence is ~5.6%, whereas China and South America reported much higher incidence rates. Risk Factors associated with ICP development include maternal age at the time of pregnancy (< 25; ≥35 years), low maternal body weight prior pregnancy, insufficient maternal weight gain during pregnancy as well as low maternal education level (see Figure 1). Furthermore, the prior use of oral contraceptives, multiple pregnancies, or assisted reproductive therapy are associated with increased ICP risk as well—which is partially attributed by the cholestatic effects of estrogen (5). Finally, women with pre-existing liver disease (including hepatitis B, C or cholelithiasis), nutritional deficiencies (selenium, vitamin D), genetic disposition, such as mutation of *ABCB4*, *ABCB11*, *ATP8B1*, *ABCC2*, *NR1H4*, and *TJP2*, maternal or family history of ICP as well as certain ethnic group affiliations show an increased risk for the development of ICP (1, 4, 6).

### 1.2 Aetiology

Most researchers agree that increased estrogen and progesterone levels as well as genetic and environmental factors contribute to the exacerbation of the physiological procholestatic profile during pregnancy, while the specific contribution of the gut microbiome to the aetiology and pathophysiology of ICP remains insufficiently investigated and not yet fully understood (1, 7). Notably, bile acids (BA) have a central role in ICP development.

BA are amphipathic molecules, physiological detergents, which are typically conjugated to glycine or taurine (8). The BA composition depends on its biotransformation via the gut microbiome and the resulting enterohepatic circulation (9). BA exert their function as endogenous modulators, signal molecules, of membrane or nuclear receptor, in absorption of dietary lipids and fat-soluble vitamins, cholesterol homeostasis (10), enterohepatic circulation regulation (via FXR signaling), host immune system regulation (modulation Th17/T-reg balance) (11, 12), excretion of toxic substances, gut microbiome regulation and balancing lipid metabolism and energy consumption (13).

**Figure 2:**
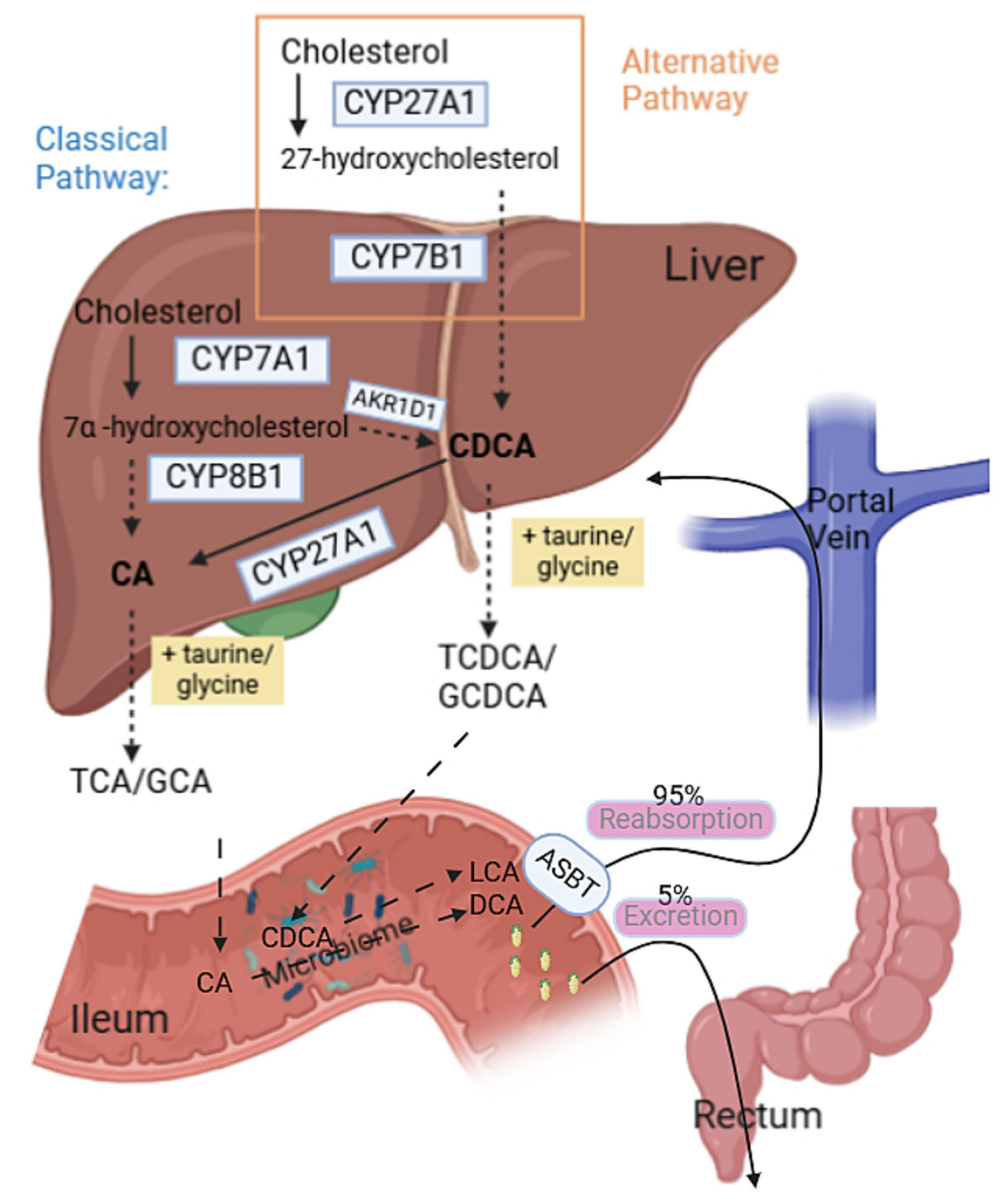
Enterohepatic circulation. Bile acids (BA) are synthesized in the liver via the classical and alternative pathway, conjugated with taurine or glycine, and secreted into the intestines. There, the resident gut microbiota deconjugate the BAs. Approximately 95% of BAs are reabsorbed and recirculated via enterohepatic circulation, while the remaining 5% are excreted. (*CYP27A1* cholesterol 7α-hydroxylase, rate limiting enzyme of the classical pathway; *CYP7B1* sterol 12α-hydroxylase, determines the ratio of CA:CDCA; *CYP7A1 s*terol 27-hydroxylase, key enzyme of the alternative pathway; *CYP8B1* oxysterol 7α-hydroxylase, works in the alternative pathway; *CA* cholic acid, primary bile acid; *CDCA* chenodeoxycholic acid, primary bile acid; *TCA* taurocholic acid; *TCDCA* taurochenodeoxycholic acid; *GCA* glycocholic acid; *GCDCA* glycochenodeoxycholic acid; *LCA* Lithocholic acid, secondary bile acid, CDCA-derived, formed through microbial enzymatic activity; *DCA* deoxycholic acid, secondary bile acid, CA-derived, formed through microbial enzymatic activity; ASBT apical sodium-dependent bile acid transporter, localized in ileum, mediates reabsorption of BAs back into the portal circulation).

BA are synthesized via the classical (90%) or the alternative pathway (10%) as summarized in Figure 2. In the classical pathway, the primary bile acids CDCA and cholic acid (CA) are synthesized in the liver. This process is regulated by the enzymes cholesterol-7alpha-hydroxylase (CYP7A1), which is rate limiting, and sterol-27-hydroxylase (CYP27A1). The primary bile acids then bind taurine or glycine in a 1:3 ratio (1, 14). The ATP-dependent bile salt export pump (BSEP) exports them to the gallbladder for storage. Following a postprandial signal from cholecystokinin (14), the BA are excreted into the duodenum. In the intestine, microbial enzymes deconjugate primary BA to secondary BA’s deoxycholic acid (DCA) and lithocholic acid (LCA) and other secondary BA’s (1, 15), which are typically more hydrophobic and some even cytotoxic (16). In the distal ileum, 95% of the BA are reabsorbed via the apical sodium dependent bile acid transporter (ASBT) then pass through the portal vein back to the liver, the rest is excreted. Thus, only 500 mg of BA per day are synthesized *de novo* (14, 17).

In the alternative pathway, the enzyme sterol-27-hydroxylase (CYP27A1) hydroxylates cholesterol extrahepatically. This metabolite is then absorbed into the liver, where the largest proportion is converted to chenodeoxycholic acid (CDCA) (1).

FXR is a nuclear receptor, expressed in the liver and intestine among other locations, which acts as master regulator for BA homeostasis (18). CDCA, a primary BA, is the most potent natural FXR activator (19). Activation of FXR signals negative feedback for BA synthesis and positive feedback for BA efflux (20). FXR activation induces Fibroblast growth factor 19 (FGF19) secretion, which then activates Fibroblast growth factor receptor 4 (FGFR4) that inhibits the expression of CYP7A1, the rate limiting enzyme of BA synthesis, in the hepatocyte. FXR activation also increases BSEP expression, that exports BA into the bile caniculi, and inhibits the expression of sodium taurocholate cotransporting polypeptide (NTCP), which reimports recirculated BA into the hepatocyte (18). The overall effect of FXR is a low intrahepatic BA level, preventing cholestasis (21, 22).

In over 15% of the ICP patients, a genetic mutation in the hepatocellular transport protein Multidrug Resistance Protein 3 (ABCB4) was reported (9). Other genetic mutations affect the farnesoid X receptor (FXR), a bile acid (BA) receptor (*NR1H4 gene*) respectively bile salt export pump (BSEP) transporter (*ABCB11 gene*)—a BA transporter (for details see Table 1). Several studies highlight the importance of 3β sulphated progesterone metabolite (PM5S) – a functional FXR antagonist disrupting the BA homeostasis (23). Environmental factors including nutrition also contribute to the pathogenesis of ICP (24). Importantly, several studies point towards the association between the gut microbiome and ICP (3).

**Table 1:** Transporters of the enterohepatic circulation.

Gene	Name	Abbreviation
*ABCB4*	Multidrug resistance protein 3	MDR3
*ATP8B1*	familial intrahepatic cholestasis protein 1	FIC1
*ABCC2*	Multidrug resistance-associated transporter 2	MRP2
*ABCB11*	Bile salt export pump	BSEP
*ABCG5*	ATP-binding cassette transporter G5	STSL2
*ABCG8*	ATP-binding cassette transporter G8	STSL1
*NR1H4*	Farnesoid X receptor	FXR
*SLC10A2*	Apical sodium-dependent transporter	ASBT
*SLC51A*	Organic solute transporters alpha	OST alpha
*SLC1B*	Organic solute transporters beta	OST beta
*FABP6*	Ileal bile acid-binding protein	IBABP
*SLC10A1*	Na + −taurocholate cotransporting polypeptide	NTCP
*TJP2*	Tight junction protein ZO-2	PFIC4

The enterohepatic circulation of bile acids involves several key transporters that regulate the uptake, secretion, and recycling of bile acids between the liver, intestine, and bloodstream. These transporters are crucial for maintaining bile acid homeostasis. A mutation in genes encoding these transporters can predispose individuals to cholestatic liver diseases, such as intrahepatic cholestasis of pregnancy (ICP).

### 1.3 Diagnosis and clinical manifestation

The ICP diagnosis is based on detection of elevated serum markers during pregnancy including total bile acid level/concentration (TBA), liver transaminases levels (ALT), aspartate aminotransferase (AST) and *γ*-glutamyltransferase (GGT) in combination with clinical manifestation (4). The ICP diagnosis is made after exclusion of other dermatological and liver diseases or drug-induced cholestasis (4). Most researchers agree that ICP is diagnosed on the basis of a TBA concentration above 10 μmoL/L in combination with maternal pruritus, while severe ICP is diagnosed with fasting TBA levels above 40 μmoL/L (1). The onset of the pruritus should be during pregnancy and resolve shortly postpartum (4). The recurrence rate of ICP in a subsequent pregnancy is as high as 40–70% (25).

Patients with ICP typically present with new onset of mild to severe pruritus in the absence of a rash and rarely show signs of jaundice (1, 3, 14). The itching is usually localized to the soles of the feet and palms of the hands, frequently generalizes and often worsens at night (4, 24). Other rare symptoms include pain in the upper right quadrant, loss of appetite, nausea, steatorrhea and sleep deprivation (4). In general, ICP pregnancies are associated with maternal dyslipidemia and increase the risk of gestational diabetes mellitus (7). Interestingly, ICP patients also have a higher risk for postpartal hemorrhage, preeclampsia, hepatobiliary cancer as well as immune and cardiovascular diseases (14). Maternal BA levels positively correlate with fetal levels and severe ICP (BA levels ≥ 40 μmoL/L) is associated with adverse perinatal outcomes (7). Adverse perinatal outcomes include spontaneous or iatrogenic premature delivery, neonatal Intensive Care Unit admission, fetal distress, fetal growth restriction, fetal asphyxia, low Apgar Score, meconium-stained fluid, respiratory distress syndrome, and stillbirth (2, 3, 7, 18, 23, 26). The pathophysiology is not fully understood but in case of spontaneous prematurity, elevated sensitivity of oxytocin receptors may be triggered by elevated BA levels (4). From the fetal perspective elevated BA may trigger arrythmia, a prolonged interval between the onset of the P wave to the start of the QRS complex and abnormal calcium kinetics in the fetal heart, which lead to higher risk of stillbirth (1). Statistically the risk of perinatal complications increases 1–2% per 1 μmoL/L TBA levels (14, 25).

In summary, early and accurate diagnosis of ICP is necessary to reduce the perinatal burden and the ICP diagnosis is confirmed when maternal symptoms as well as biochemical abnormalities resolve shortly after delivery (27, 28).

### 1.4 Therapy

Despite the heterogeneous aetiology of ICP, current treatment strategies remain largely empirical, as the underlying pathophysiology is not fully understood (2). Consequently, ICP management primarily aims to reduce perinatal morbidity and mortality and to alleviate maternal symptoms (4). Ursodeoxycholic acid (UDCA) is the first-line pharmacological therapy (1); however, evidence for disease-modifying treatments is limited, highlighting the need for further investigation of therapeutic approaches.

Delivery is considered the definitive therapeutic approach for intrahepatic cholestasis of pregnancy, with the timing of delivery determined by factors such as serum bile acid levels, gestational age, and maternal-fetal risk factors (29). Delivery is the only intervention that leads to resolution of intrahepatic cholestasis of pregnancy and prevents fetal complications, including stillbirth (30). In clinical management, total serum bile acid levels are commonly used as the primary parameter to guide these decisions. When bile acid concentrations are markedly elevated (≥100 μmol/L), delivery is generally considered around 36 weeks of gestation, or at the time of diagnosis if the condition is first identified after this point. If bile acid levels are lower, the timing of delivery is typically determined on an individual basis and usually scheduled between 36 and 39 weeks of gestation (31).

## 2 Gut microbiome

The human gut microbiome is an ecosystem containing all microorganisms in the human intestine (32). It is composed of over 10^13^ microorganisms including bacteria, viruses, eucaryotes, parasites and archaea (33, 34). Sometimes referred to as a virtual organ of the human body, its interaction with the host is important for a number of physiological functions (3). These include fermentation of indigestibles (e.g., short chain fatty acid from dietary fibers), bile acid metabolism (e.g., deconjugation from 1° to 2° BA via BSH activity, hydroxylation), vitamin K synthesis, intestinal barrier protection, as well as immunomodulation (3, 10, 11, 35–37). Via the gut-liver axis, the liver is susceptible for the influence of the gut microbiome (12). The gut microbiome composition is affected by host genetics, diet, hormones, immune system and a variety of environmental factors (13, 38–41). To adapt to the changing needs of the organism, the gut microbiome composition changes. Especially in pregnancy, this process is important in order to optimize maternal metabolism for fetal growth and development (1).

A drastic shift or imbalance in gut microbiome composition and activity, leading to dysbiosis, can induce gastrointestinal diseases, even in non-predisposed individuals (2, 42, 43). Dysbiosis is associated with intestinal barrier dysfunction, increased intestinal permeability and systemic inflammation (26). Dysfunction of intestinal barrier are involved and contributed to establish several metabolic diseases, including type 2 diabetes mellitus (44) inflammatory bowel disease (45) and metabolic syndrome (28).

### 2.1 Gut microbiome and pregnancy

To adapt to the changing needs of an organism, the gut microbiome composition changes. Especially in pregnancy, this process is important in order to optimize maternal metabolism for increased needs such as fetal growth and development (1). A shift or imbalance in gut microbiome composition and activity—dysbiosis contributes to pregnancy-related conditions, including gestational hypertension, preeclampsia, and gestational diabetes (1).

In the context of ICP the gut microbiome affects BA synthesis and metabolism via gut-liver axis and enzymatic activities (46). The disease development is characterized by inflammation, oxidative stress, a decreased bile flow through the liver and a disrupted excretion, resulting in accumulation of toxic BA (27, 47). Due to their hepatotoxicity, aminotransferases, bilirubin and alkaline phosphatase are released from hepatocytes and accordingly are elevated in the serum (26). Maternal BA may cross the placenta and enter fetal circulation increasing the BA concentration in the fetal compartment – at the end putting the fetus at risk (1).

This systematic literature review aims to critically synthesize the complex, bidirectional association between the gut microbiome and ICP, specifically highlighting mechanistic insights and emerging therapeutic targets derived from both human and animal studies, to inform future research and clinical strategies.

## 3 Methods—systematic literature review

We conducted a comprehensive literature search using the PubMed Central (PMC) database. Two independent reviewers (FD and MM) performed a structured literature search on PubMed using the following search string: (gut microbiome OR gut microbiota) AND (intrahepatic cholestasis of pregnancy OR hypercholanemia in pregnancy OR ICP). The inclusion of synonyms in the search term was intended to maximize the retrieval of potentially eligible papers. The keywords were deliberately chosen to cover the entire spectrum of relevant literature on intrahepatic cholestasis of pregnancy without capturing many irrelevant studies. An initial exploratory search was conducted in PMC on February 14, 2024, followed by comprehensive searches in MEDLINE, Embase, and Google Scholar to ensure the broadest possible coverage of various publication channels and indexing systems. Throughout this process, we adhered to the international PRISMA guidelines for systematic reviews (48).

Studies were included based on their relevance to the research topic and their classification as either “original research article” or “review.” We included both animal and human studies. Therefore, we excluded “conference abstracts,” “meeting transcripts,” and papers not aligned with the subject of interest. Given the limited availability of studies employing human models, we also included research conducted on animal models to broaden the scope of evidence. Studies involving animal models are discussed separately in a dedicated subsection of the Results section.

The initial search yielded 28 publications. After title and abstract screening, 25 full-text articles were assessed for eligibility. Following the exclusion of four articles, 21 articles met the inclusion criteria, comprising 3 review papers and 18 articles. To manage and analyze the retrieved data, we constructed a comprehensive Excel database, which we continuously supplemented with bibliographic details, key findings, critical reflections, and direct links to the full texts during the review and manuscript preparation process.

## 4 Results

**Figure 3:**
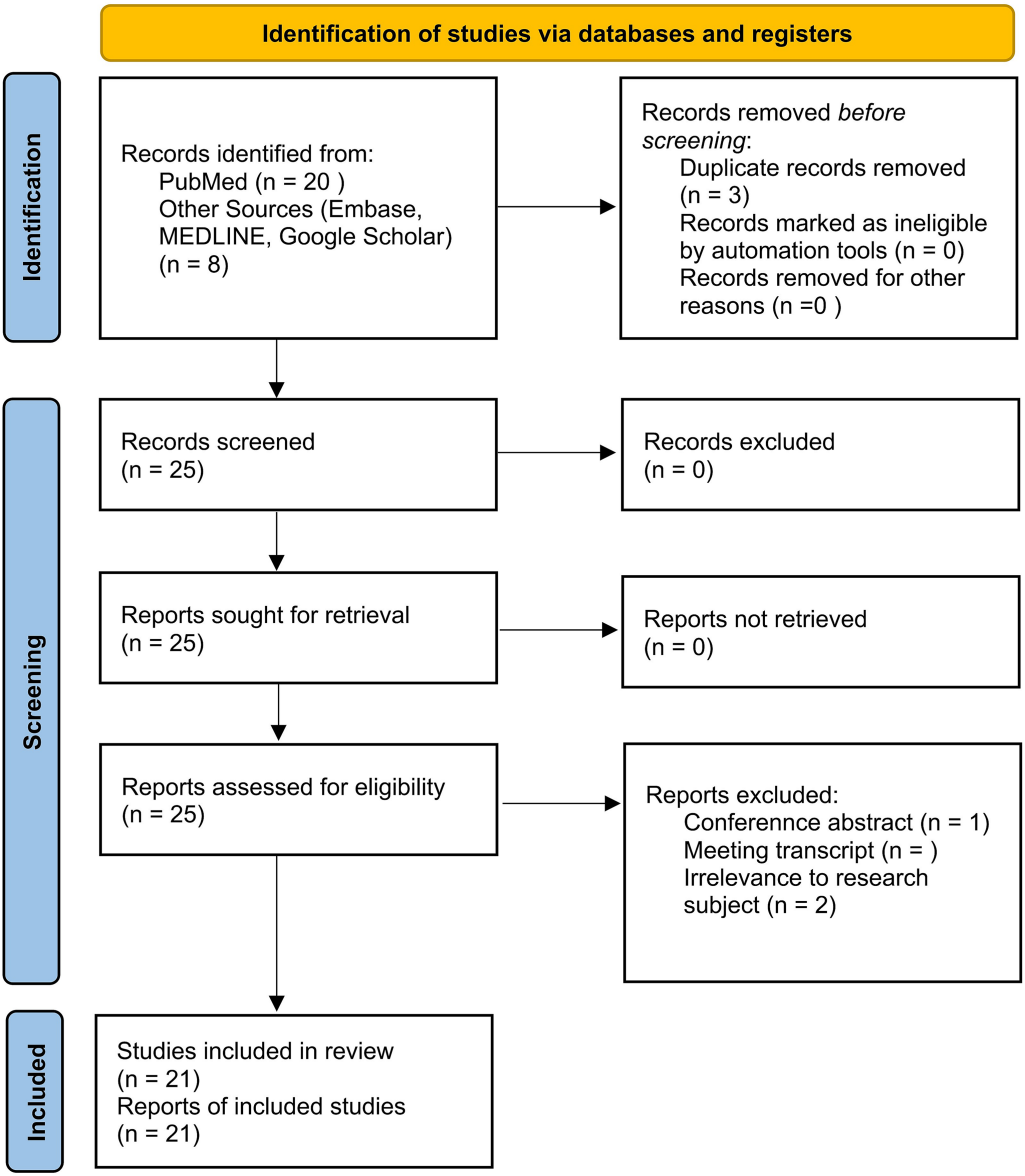
Screening process according to international PRISMA guidelines. During the screening process we identified 28 publications, which included both animal and human 881 studies. Finally, we included 21 studies in the systemic review.

We included a total of 21 articles, 3 of which are reviews and 18 of which are original research articles. The selection process is summarized in Figure 3. We identified several pregnancy-related diseases, which are associated with an imbalance of the gut microbiome (2). The following sections review the current state of research on the relationship between ICP and the gut microbiome.

### 4.1 Gut microbiome changes in pregnancy

Multiple studies have demonstrated significant alterations in the gut microbiome between ICP patients and healthy pregnant women (14). The gestational gut microbiome is predominantly composed of four phyla: *Firmicutes*, *Bacteroidetes*, *Actinobacteria* and *Proteobacteria* (24). From first to third trimester, there is an overall increase *in Proteobacteria and Actinobacteria* and a decrease in the richness of the gut microbiome (49). In the second trimester, the phylum *Firmicutes* is dominant, bacterial genera *Streptococcus, Megasphaera* and *Bacteroides* are abundant (49, 50). In the third trimester, members of the *Enterobacteriaceae* family and *Streptococcus* genus are dominant (24, 49). Importantly, the gut microbiome composition in the third trimester, when ICP typically occurs, differs significantly from the first trimester (46).

In ICP, alterations of the gut microbiome may contribute to disturbed bile acid homeostasis. Compared with healthy individuals, ICP patients exhibit a reduced relative abundance of Firmicutes (26), a phylum involved in short-chain fatty acid production and regulation of key enzymes in bile acid metabolism (1, 51). This reduction may impair bile acid metabolism and promote a cholestatic state (46). In contrast, physiological pregnancy is characterized by an increased Bacteroidetes/Firmicutes ratio, with Bacteroidetes expressing bile salt hydrolase, thereby enhancing bile acid deconjugation and synthesis and potentially explaining mild hypercholanemia in healthy pregnancy (9, 17, 52). Lastly, alpha and beta diversities, the species diversities within and between samples, are changing during a physiological pregnancy as well. Reduced alpha diversity and increased beta diversity in late gestation, may further modulate bile acid metabolism (47).

### 4.2 Gut microbiome changes in ICP

Most included studies reported a significant difference in the gut microbiome composition between healthy and ICP pregnancies. Only one study did not confirm a difference in the main composition of the gut microbiome but detected differences in abundances on several taxonomical levels (24). The gut microbiome modifies BAs and other metabolites (e.g., SCFA, tryptophan) and thus changes the serum metabolome in ICP (2, 14). A depletion of SCFA and butyrate-producing bacteria and an increase in BA-metabolizing bacteria in ICP patients was reported. Alterations in metabolism pathways could disturb the gut microbiota composition (26). A fecal sample study concluded that the abundances of rare bacterial populations such as *Blautia* and *Citrobacter* are more frequent in ICP patients (24). ICP model rats had a significantly reduced biodiversity of intestinal microorganisms, lower fecal CDCA levels as well as altered gut microbiome and BA pool composition in comparison to the control group (18).

In summary, a significant gut microbiome composition, characterized by a reduced biodiversity, shift in bacterial phyla and colonization of rare bacterial species, can be observed in ICP. This characteristic ICP gut microbiome displays a different serum metabolome, distinguished by a SCFA and butyrate depletion compared to healthy controls.

#### 4.2.1 Interaction between the gut microbiome and bile acids

The altering gut microbiome composition may interfere with BA homeostasis through the expression of enzymes, via the gut-liver axis, and may be the origin of the subsequent hypercholestatic state (46). Further, a dysregulated BA secretion leading to BA accumulation, may affect the gut microbiome composition—the resulting disrupted immune response could act as a disease trigger for ICP (26). The gut microbiome composition is affected by other factors as well. For example, the pregnancy-specific elevation in sex hormones estrogen and progesterone might exert microbial selection pressure on certain gut bacteria, leading to dysbiosis in susceptible individuals (2, 53, 54). Microbial homeostasis can be regulated by BA, by limiting the growth of bile acid sensitive bacteria (55, 56). In a cholestatic state, where BA secretion is prevented, BA-sensitive bacteria can grow excessively, which leads to dysbiosis and a microbial translocation across the small intestine (57, 58).

In summary, the interaction between the gut microbiome and BAs seems to contribute to the development of the ICP-specific dysbiosis, which in turn deteriorates with the BA metabolism increase, resulting in vicious cycle (46).

### 4.3 Bacterial abundance in ICP

The following section aims to summarize the change in bacterial abundance occurring in ICP and is arranged by taxonomical levels and levels of severity. An overview of reported microbiome changes during ICP is provided in Table 2.

**Table 2:** Changes in microbial abundances in ICP.

Taxonomy	Increased	Decreased	Citation	
Phylum	*Bacteroidetes*	*Firmicutes*	Li GH, et al. (26) and Ovadia C, et al. (9)	
	*Proteobacteria*		Zhan Q, et al. (25), Li X, et al. (46)	
	*Bacteroidetes*, *Proteobacteria*, *Deferribacteres*, Verrucomicrobia*	*Actinobacteria*, *Firmicutes*, *Tenericutes*, *Spirochaetes*, Patescibacteria*	Li Z, et al. (47)	
	Proteobacteria**	*Firmicutes*, Bacteriodetes**	Li X, et al. (46)	
Class	*Bacilli*, *Gammaproteobacteria*		Li R, et al. (24)	
	Gammaproteobacteria**		Li X, et al. (46)	
Order	*Enterobacteriales*, *Lactobacillales*		Li R, et al. (24)	
	Enterobacteriales**		Li X, et al. (46)	
Family	*Enterobacteriaceae*, *Leuconostrocaceae*, *Streptococcaceae*		Li R, et al. (24)	
	*Lactobacillaceae*		Zhan Q, et al. (25)	
	*Enterobacteriaceae*, Staphylococcaceae**	Ruminococcaceae**	Li X, et al. (46)	
Genus	*Blautia*, *Citrobacter*		Li R, et al. (24)	
	*Parabacteroides*, *Bilophilia*, *Bacteroides*, *Escherchia*/*Shigella*	*Faecalibacterium*, *Blautia*, *Bfidiobacterium*	Li GH, et al. (26)	
	*Flavonifractor*, *Atopobium*, *Turicibacter*, *Parabacteroides*, *Lactobacillus*, *Escheichia*/*Shigella*	*Megamonas*	Zhan Q, et al. (25)	
	*Bacteroides fragilis*, *Klebsiella pneumoniae*, *Klebsiella variicola*, *Klebsiella quasipneumoniae*, *Weisella confusa*, *Citrobacter youngae*, *Enteroacter clocae*	*Coprococcus_catus*	Tang B, et al. (2)	
	*Bilopphila*, *Parabacteroides*, *Shigella*	*Faecalibacterium*	Huang X, et al. (28)	
	*Ruminoclostridium*, Bilophilia*		Li Z, et al. (47)	
	*Escherichia_Shigella*, *Olsenella*, Turibacter**		Zhan Q, et al. (25)	
	*Escherichia_Shigella*, *Lachnochlostridium*, *Ruminococcus*, *Erwinia*, Staphylococcus**		Li X, et al. (46)	
		*Bifidobacterium*	Tang M, et al. (14) (Review)	
Species	*Streptococcus luteciae*, *Clostridium methylpentosum*		Li R, et al. (24)	
	*Bacteroides fragilis*, *Klebsiella pneumoniae*, *Klebsiella variicola*, *Citrobacter youngae*, *Enterobacter clocae*		Tang M, et al. (14)	
		*Roseburia intestinalis*	Sun H, et al. (18)	
		*Eubacterium hallii*	Liu ZZ, et al. (3) (Review)	

* In ICP rat model; ** In severe ICP cases. This table aims to summarize the change in bacterial abundance occurring in ICP and is arranged by taxonomical levels and levels of severity. It gives an overview of the described gut microbiome alterations, increased and decreased abundances, during ICP pregnancy.

#### 4.3.1 Phylum level

At phylum level ICP mouse studies confirm a decrease of relative abundance of the *Firmicutes* and an increase of the *Bacteroidetes* (26, 47). Bacteroidetes exhibit bile salt hydrolase (BSH) activity and deconjugate primary bile acids (BAs), promoting the formation of secondary BAs such as deoxycholic acid (DCA) and lithocholic acid (LCA) (14, 27). These secondary BAs have lower reabsorption rates and modulate FXR and TGR5 signaling (2). Reduced FXR activation leads to decreased FGF19 expression, resulting in disinhibition of hepatic BA synthesis via CYP7A1 (52, 59, 60). In addition, LCA and DCA are potent TGR5 agonists and exhibit increased cytotoxicity, potentially exacerbating ICP (14, 15).

Overall, ICP mouse models show increased abundances of *Bacteroidetes*, *Proteobacteria*, *Deferribacteres* and *Verrucomicrobia*, alongside reduced *Actinobacteria*, *Firmicutes* and several minor phyla (47). The phylum *Proteobacteria*, which displays increased abundance in ICP, is associated with various inflammatory and metabolic conditions (1).

#### 4.3.2 Class, order and family levels

At higher taxonomic resolution, ICP is associated with increased abundances of the classes *Bacilli* and *Gammaproteobacteria,* along with increased abundances of orders *Enterobacterials* and *Lactobacillales* (24). At the family level, higher abundances of *Enterobacteriaceae*, *Leuconostocacae* and *Streptococcaceae* were noted (3, 24).

#### 4.3.3 Genus level

At the genus levels the bacterial abundance in ICP are associated with modified BA metabolism. Reduced abundance of *Megamona*s and enrichment of *Flavonifractor, Atopobium, Turicibacter, Parabacteroides, Lactobacillus* and *Escherichia*_S*higella* have been observed in ICP patients (25). Increased abundance of genera *Blautia, Citrobacter* and *Streptococcus* and enrichment of the genera *Bilophila* and *Ruminococcus*, further support disturbed BA transformation (47).

SCFA producing bacteria, such as genera *Faecalibacterium*, *Blautia* and *Bifidobacterium*, are depleted in ICP patients (3). These taxa are associated with gut barrier integrity, glucose metabolism and anti-inflammatory effects (14). In contrast, bile-tolerant and potentially pro-inflammatory genera such as *Parabacteroides* and *Bilophila* are enriched and contribute to secondary BA production and inflammatory signaling (26). Collectively, changes of the beneficial genera such as depletion of SCFA producing and the enrichment of potentially harmful (bile-tolerant) microbes is contributing to ICP. The resulting modifications include BA metabolism and pro-inflammatory environment (1).

#### 4.3.4 Species level

At the species levels the higher abundance of *Bacteriodes fragilis (B. fragilis)* in ICP patients contributes substantially to modified microbial profile (27). This species exhibits BSH activity and indirectly supresses FXR signaling, thereby promoting excessive BA synthesis and impaired BA homeostasis (14). In line with this notion, ICP stool transplanted mice developed ICP-like phenotypes, including elevated BA, liver damage, and lower FGF15 levels (2). Notably, *B. fragilis* correlates with fetal birth weight negatively and in contrast positively with TBA levels. The abundance of *B. fragilis* contributes to ICP development (14). Other changes include a higher abundance of species *Streptococcus luteciae* (24) and a depletion of the SCFA-producing species *Eubacterium hallii* (26).

In summary, ICP is characterized by gut microbiome dysbiosis with an overall shift towards the *Bacteroidetes* and *Proteobacteria and depletion of Firmicutes*, particularly SCFA-producing taxa (26). These alterations exacerbate disturbed BA metabolism and inflammation, reinforcing a vicious cycle that contributes to ICP pathogenesis.

### 4.4 Abundance changes and ICP severity

The extent of gut microbiome alterations appears to be associated with ICP severity. In severe cases (TBA > 40 μmoL/L), microbial changes are more pronounced compared to moderate cases (10–39 μmoL/L) and healthy controls. At the phylum level, severe ICP is characterized by a decrease in *Firmicutes* and *Bacteroidetes* and an increase in *Proteobacteria* (46). *Proteobacterial* expansion, is a sign for epithelial dysfunction and allows pathogenic bacteria to invade in bile ducts (46, 61). In addition, the reduction of *Firmicutes* may contribute to diminished anti-inflammatory signaling related to secondary bile acid metabolism (62, 63). On the family level, severe ICP is associated with lower relative abundance of *Ruminococcaceae* and higher levels of *Enterobacteriaceae* and *Staphylococcaceae*. The *Ruminococcaceae* family is known to have a protective role in liver disease (46) and depleted in other liver diseases, including non-alcoholic fatty liver disease and cirrhosis (64). Thus, the depletion of *Rumionococcaceae* may contribute to ICP pathogenesis similarly as in other liver diseases (46). On the genus level, *Escherichia_Shigella, Olsenella, Lachnoclostridium, Ruminococcus, Erwinia, Staphylococcus* and *Turibacter* are enriched in severe ICP (25, 46). Notably, *Escherichia_Shigella* shows a positive correlation with TBA levels and may be involved in a feedback mechanism that parallels increasing cholestasis severity (46, 65, 66). Additionally, *Bacteriodes fragilis* is more abundant in severe compared to moderate ICP (2).

In contrast, moderate ICP presents with less pronounced microbial alterations, and the overall composition more closely resembles that of healthy controls (46). An increased abundance of the genus *Flavonifractor* has been reported in moderate cases (25), but overall shifts are milder than in severe ICP.

In summary, more severe ICP is associated with more distinct and extensive alterations of the gut microbiome, suggesting a relationship between microbial imbalance and disease severity.

### 4.5 Metabolites and pathways in ICP

The gut microbiome contributes to pathophysiological processes through metabolites such as trimethylamine oxide, BA and SCFA. Overall, the serum metabolome in ICP differs significantly from healthy controls, and in severe ICP cases, stool metabolite profiles are particularly distinct (46). These findings suggest that altered metabolic pathways may contribute to microbial imbalance. Indeed, pathways related to primary BA biosynthesis and taurine and hypotaurine metabolism are enriched in ICP patients (26).

Animal models further demonstrate molecular alterations associated with ICP (18). Impaired intestinal barrier function, reflected by reduced expression of Occludin, Claudin-1, and tight junction protein 1, is linked to liver inflammation and increased pro-inflammatory cytokines (IL-6, IL-1β, TNF-*α*). In the liver, CYP7A1 expression is increased, whereas FGFR4, SHP, and BSEP are decreased. In the ileum, reduced SHP, FXR, and FGF15 levels lead to impaired FXR-FGF15 (human FGF19) signaling. This inhibition results in increased BA synthesis, due to CYP7A1 induction, and decreased BA export (18).

#### 4.5.1 Butyrate and hypoxanthine metabolism

In severe ICP cases, the fecal metabolite composition is complex and significantly altered. BA, formate, succinate levels are increased and the butyrate and hypoxanthine levels decreased (46). The increased formate levels correspond to the *Acinetobacter* enrichment, which is an important genus in the formate metabolism. Butyrate, on the other hand, is a protective factor for gastrointestinal disease (26). Butyrate reduces pH in the colon, regulates pathogenic bacteria growths, regulates intestinal immunity and serves as energy source for epithelial intestine cells (67). Four bacterial genera were associated with a lower risk for ICP: *Dialister, Erysipelatoclostridium, Eubacterium* and *Ruminococcus.* Not surprisingly, all of them are butyrate-producing genera. Butyrate is important for the gut barrier function, immunomodulation as well as anti-inflammation (27).

Also, the butyrate and hypoxanthine metabolism are strongly associated with gut microbiome. Finally, the hypoxanthine metabolism is depleted in severe ICP. The species *Ruminococcus gnavus*, *Lachnospiracea FCS020*, *Lachnospiraceae NK4A136* play a major role in the hypoxanthine metabolism (46).

#### 4.5.2 BA and tryptophane metabolism

The BA pool is altered in ICP. ICP patients have a reduced proportion of unconjugated BA, increased proportion of taurine and glycine conjugates (68) and the CA/CDCA ratio is higher in ICP pregnancies than in healthy pregnancies (69). CA is only a weak FXR activator, compared to CDCA, resulting in lower FXR activity which reinforces the procholestatic state (1, 19). Other changes in the BA composition include the conjugation of BAs. In ICP, toxic taurine conjugate levels are higher, whereas in healthy pregnancies glycine conjugate levels are increased (70). Furthermore, the abundance of the genera *Atopobium, Turibacter, Flavonifractor, Escherichia_Shigella* and *Olsenella* correlated positively with TBA, the abundance of family *Ruminococcacea*, genus *Megamonas*, and species *Eubacterium coprostanoligenes* negatively, making these genera markers of severity (25, 46).

The gut microbiome influences tryptophane metabolism pathways, including kynurenine, indole and serotonin derivate metabolites (14). In ICP patients, kynureine, 3-hydroykynureine, 2-oxoadipic acid and indole metabolite levels differ significantly from healthy women. Additionally, valeric acid, which is a gut microbial metabolite, is reduced in ICP patients. In contrast pantothenate (vitamin B5) levels were increased in ICP patients (71). Thus, the abnormal tryptophan metabolism may be associated with ICP microbial flora (14, 47). Other pathways contribute to development of ICP as well. For example, a depletion of short-chain fatty acids SCFA producing bacteria, including genera *Faecalibacterium, Blautia and* species *Eubacterium hallii*, has been noticed (26). *Blautia* have a beneficial role for the glucose metabolism (72).

Together, ICP-associated microbial composition changes influenced metabolic pathways, particularly those related to BA metabolism, inflammation and lipid metabolism. This is consistent with the altered BA profiles and altered liver enzymes observed in ICP (1).

### 4.6 ICP therapeutics targeting the gut microbiome

Despite the heterogenous ICP aetiology, several potential treatment approaches exist. ICP treatment is empirical as the aetiology is not fully understood (2). Therefore, ICP management focuses on the reduction of perinatal morbidity and mortality and the relief of maternal symptoms (4). Ursodeoxycholic acid (UDCA), a secondary BA, is the first line pharmacolgical therapy (1). UDCA acts primarily through modulation of bile acid composition and enhancement of hepatobiliary secretion. By enriching the bile acid pool with less toxic species and promoting choleresis, UDCA reduces both maternal symptoms and serum bile acid concentrations. Additionally, UDCA exhibits cytoprotective effects on hepatocytes and modulates immune responses. When pruritus persists, rifampicin or systemic antihistamines may be considered. Due to the risk of fetal events, timing the delivery (in some cases premature delivery) is an option. The timing of delivery depends on BA concentration and gestational age. Induction of labor is recommended between 34 + 0 and 36 + 6 weeks of gestation if the BA level increase >100 μmoL/L and > 37 + 0 if the BA level is <100 μmoL/L (4, 73).

Microbiota-targeted interventions represent a promising therapeutic strategy currently under investigation.

#### 4.6.1 Ursodeoxycholic acid

Ursodeoxycholic acid (UDCA), a naturally occurring BA derivate with anti-cholestatic effect in humans, is the first line pharmacological therapy at present and is commonly used off-label for ICP treatment (1, 4). UDCA treatment reduced maternal itching (74) and changed maternal BA pool composition (9). After UDCA admission, ICP patients displayed a higher fecal LCA, unconjugated BA, serum FGF19 level and a correspondingly reduced 7-alpha-Hydroxy-4-cholesten-3-one (C4) level, a metabolite in the cholesterol synthesis which reflects the BA synthesis activity. Thus, a reduced C4 reflects a lower BA synthesis rate which resulted from the increased negative feedback via FGF19 elevation (9). UDCA is not a potent FXR agonist itself, but the gut microbiome enzymatic activity deconjugates it to secondary bile acids LCA and CDCA (75). These secondary BAs are metabolically active and initiate processes that ultimately lead to the benefits of UDCA treatment. UDCA treatment is linked to a decreased relative abundance of the *Firmicutes* phylum and increased abundance of the *Bacteroidetes* phylum. *Bacteroidetes* are a phylum with high BSH activity. Hence, *Bacteroidetes* deconjugate primary BAs, which then are transformed to secondary BAs, functional FXR agonists. The result of this process is higher enterohepatic feedback, via FGF19, which leads to a lowered BA synthesis. Notably, women with a higher *Bacteroidetes*: F*irmicutes* ratio prior to treatment are more susceptible to UDCA (9).

#### 4.6.2 Potential new therapeutics—animal studies

##### 4.6.2.1 Reservatrol

Reservatrol, a natural polyphenolic compound (76), was examined in ICP rat models. Reservatrol may reverse the induced dysbiosis, characterized by an increase of the phylum *Actinobacteria*, the genera *Ruminiclostridium* and *Bilophila*, After Reservatrol treatment, the phyla Actinobacteria, *Synergistetes* and *Chloroflexi* increased, the phylum *Tenericutes* decreased. Additionally, TBA and ALT levels could be reduced with Reservatrol. In conclusion, Reservatrol improves gut microbiota dysbiosis and laboratory markers in ICP rat models (47). However, more research including clinical studies in humans are needed to confirm the efficacy and safety of reservatrol admission in ICP.

##### 4.6.2.2 Obeticholic acid

Obeticholic acid (OCA) is a semisynthetic BA, functioning as a FXR agonist (77), which improved fetal BA profile in an ICP mouse model (7). OCA is also used in primary biliary cholangitis (PBC) treatment (78). OCA activates FXR leading to amelioration of fetal hypercholanemia and dyslipidemia, as FXR is an important modulator of lipid metabolism and BA synthesis, transport and excretion. OCA could ameliorate fetal hypercholanemia without impact on the maternal hypercholanemia as well as maternal and fetal dyslipidemia. The fetal hypercholanemia was reduced by a decrease in fetal hepatic CYP7A1 expression and upregulation of placental transporters (e.g., MRP2, OATP1B2), which contribute to fetal BA excretion across the placenta. The administration of OCA repressed maternal hepatic CYP7A1, the BA synthesis rate limiting enzyme, via ileal FXR activation resulting in FGF15 (in human FGF19) secretion in the portal circulation. In addition, OCA-fed mice display a lower relative abundance of the *Bacteroidetes* phylum (7).

##### 4.6.2.3 Lactobacillus rhamnosus

The therapeutic effect of *Lactobacillus rhamnosus* (LGG) was examined in ICP-mouse models. LGG administration lowered serum and liver BA levels effectively, partly explained by an increased expression of BA transporter BSEP, which transports BA into the bile caniculi, and Multidrug Resistance Protein 3 (MRP3), which is important for basolateral BA export. Also, LGG administration increased FXR activation in the intestine and liver, attaining a BA synthesis inhibition via the upregulation of FGF15 (respectively FGF19 in humans). Furthermore, LGG decreased pro-inflammatory cytokine expression and reduced collagen deposition and portal fibrosis histologically. Secondly, LGG pretreatment in the ICP mouse model was investigated. LGG pretreated mice, prior to generating CA + PM5S induced ICP mouse models, had lower BA, bilirubin, liver enzyme and proinflammatory cytokine mRNA expression levels compared to the non-pretreated group. The benefits of LGG to haptic FXR activation and BSEP upregulation was noted and suggest that probiotic administration in ICP management is potentially able to intervene in the ICP pathomechanism via gut microbiome-mediated FXR activation (23).

##### 4.6.2.4 Roseburia intestinalis

*R. intestinalis* is a gram positive butyrate-producing species, belonging to the phylum *Firmicutes. R. intestinalis* is involved in intestinal barrier homeostasis, preventing LPS leakage from the intestine, and decreases liver inflammation. It has shown therapeutic effects in inflammatory bowel disease (IBD), atherosclerosis and the metabolic syndrome. *R. intestinalis* abundance is lower in ICP pregnancies (18). In an ICP rat model study, ICP fecal transplants to pseudo-germfree healthy pregnant rats resulted in an increase in serum BA levels. These results suggest that the dysbiotic ICP microflora is one of the triggers of ICP development (18). Furthermore, *R. intestinalis* transplantation resulted in a serum decrease in BA, LPS and CYP7A1 protein levels, an increase in serum CDCA levels, intestinal barrier proteins, immune balance and an ICP phenotype improvement via the FXR receptor and FGF15 signaling pathway. Overall, it caused an increase of BA transport and a decrease of hepatic BA synthesis. The researchers confirmed their result by demonstrating the reversion of benefits after applying Z-Gu, a global FXR inhibitor (18).

##### 4.6.2.5 Chenodeoxycholic acid

CDCA, a primary BA, can moderate the communication between the gut microbiome and host metabolites (79). CDCA is the most potent natural FXR activator (19). It has a protective role in ICP development (80). CDCA administration in early pregnancy improved embryo implantation and effected a reduced inflammation, oxidative stress, insulin resistance and changed the fecal metabolome in sow and rat studies (79).

## 5 Discussion

Research interest in the gut microbiome and its role in various diseases has grown significantly over the past decade. However, the etiopathogenesis of the pregnancy-specific condition ICP remains unclear, and there is still a lack of evidence-based therapeutic interventions. Against this backdrop, increasing attention is being paid to the potential role of the gut microbiome, as a more detailed understanding of its functional and mechanistic involvement in ICP is essential for guiding future developments. In particular, the identification of microbiome-associated changes could reveal new pharmacological targets and thus pave the way for innovative therapeutic strategies. The development of such microbiome-based interventions therefore remains a critical unmet clinical need that can only be addressed through a more precise understanding of the underlying mechanisms.

Studies on fecal microbiota transplantation have demonstrated that transferring ICP microbiota to mice is sufficient to induce cholestasis. The central pathophysiological mechanism involves inhibition of the FXR signaling pathway by specific bacteria such as *Bacteroides fragilis*, which modulate BA metabolism via their bile salt hydrolase (BSH) activity. This FXR inhibition leads to excessive BA synthesis and impaired hepatic bile excretion. Mendelian randomization studies confirm the causal role of certain bacterial taxa and rule out reverse causality (27). Nevertheless, it remains unclear whether microbial changes occur primarily or secondarily to hormonal/genetic predisposition (27). It is unclear which microbial interventions (specific probiotics, prebiotics, fecal microbiota transplantation) are most effective and how their timing should be optimized. The exact molecular mechanisms by which specific bacteria such as *B. fragilis* influence FXR signaling require further elucidation. Finally, the role of other microbial metabolites (besides BA) is insufficiently characterized. The considerable heterogeneity of microbial profiles among ICP patients is not fully explained and genetic, dietary, and environmental factors that determine this variability must be identified.

The mechanism and role of probiotics in ICP therapy is being investigated as well and could prove to be a cornerstone of ICP therapy or prevention in predisposed populations. In particular LGG has shown beneficial effects in ICP mouse models by reducing serum and hepatic BA levels, partly through upregulation of bile acid transporters (BSEP and MRP3). In addition, LGG enhances intestinal and hepatic FXR activation, leading to increased FGF15/FGF19 signaling and subsequent suppression of BA synthesis. These effects are accompanied by reduced inflammatory cytokine expression and attenuated liver injury and fibrosis. Overall, these findings suggest that LGG may modulate ICP pathophysiology via gut microbiome–FXR–FGF signaling pathways and could represent a promising microbiome-based therapeutic strategy. Additionally, the shift at various taxonomical levels in ICP development towards the phylum *Bacteriodetes* and away from the phylum *Firmicutes* helps to understand a great deal of how the gut microbiome composition can trigger or uphold the procholestatic state in ICP. BSH expressing bacteria are able to alter the BA metabolism sustainably and shift the BA pool to a hypercholanemic state by reducing the FXR activation due to the decreased BA reuptake rate. On the other hand, there is evidence that a hypercholanemic state and cholestasis alter the gut microbiome composition greatly. BA-dependent bacteria have excessive growth patterns in a cholestatic state, because the BAs that inhibit their growth are missing. Most likely, a complex pattern of interaction between the gut microbiome and BA pool drives the ICP development, in conjunction with other pathophysiological mechanisms. The described BA-gut microbiome axis is again regulated via the FGF19-FXR pathways. Several study results underlined the importance of this pathway for the pathological effects the gut microbiome has in the ICP disease development and manifestation.

The exact role the gut microbiome plays is not yet known, but there are several theories originating from different researchers in the field. The potential clinical importance of the microbiome is further supported by experimental data showing that stool transplants from ICP patients can induce an ICP-like phenotype in germ-free mice. This suggests that microbial composition may not merely be a consequence of cholestasis but could actively contribute to disease development. It would be interesting to know to what extent the pre-pregnancy gut-microbiome predisposes this disease. Several patterns in ICP incidence have been found geographically and ethnically. This pattern can be explained partly by the respective gene pool (accumulation of transporter mutations). Part of it may be explained by an environment-dependent, especially nutrition, microbiome composition. Furthermore, identification of pharmacological targets could follow.

The clinical relevance extends beyond the mother. ICP affects fetal BA metabolism and gut microbiota, increasing fetal susceptibility to inflammation (81). ICP offspring not only have higher incidence of postnatal jaundice and infection but can also suffer from long-term metabolic disturbances. However, the gut immunity of ICP offspring could be improved with *Lactobacillus rhamnosus* (LRX01) supplementation (81), suggesting a possible preventive or adjunct therapeutic strategy. An increased abundance *of Escherichia_Shigella* and other gram-negative bacteria characterize the gut microbiome from ICP offspring in rats (81).

Finally, the question of causal relationship between the gut microbiome and ICP risk exists. The extensively discussed changes in microbial abundances raise the question of whether these alterations are a consequence of ICP or contribute to its onset and aggravation. The causal relationship between the gut microbiome and ICP can be investigated using a bidirectional two sample Mendelian randomization (MR) design to approximate the causality between gut microbiota, as risk, and ICP, as outcome (82). Using this approach several bacteria which are associated with a greater or lower risk of ICP development were identified (27). The ICP risk was associated with a higher relative abundance of phylum *Tenericutes*, classes *Bacteroidia* and *Mollicutes* and order *Bacteroidales* (27). The phylum *Tenericutes* is associated with several pro-inflammatory cytokines, including IL-6, IL-17, TNF-alpha, which are present in ICP patients (83, 84). A lowered ICP risk was associated with a higher relative abundance of the genera *Dialister* (83), *Erysipelatoclostridium* (85), *Eubacterium* (group brachy, hallii) (86), *Holdemania*, *Ruminococcus* and *Veillonella* (27). The genera *Dialister*, *Erysipelatoclostridium*, *Eubacterium* and *Ruminococcus*, produce butyrate, and contribute to maintaining the gut barrier function, immunmodulation, energy homeostasis, and anti-inflammation (87). For those above reasons, these genera can be considered protective with respect to ICP risk (27). Finally, the direction of causality was from bacterial taxa to ICP and no reverse causality from ICP to bacterial taxa was detected (27). This idea is reinforced by a recent mouse study, which demonstrated that germ-free mice developed an ICP-like phenotype after receiving stool transplants from ICP patients. FGF15 levels and expression levels of downstream genes of FXR were suppressed in the ileum and liver of ICP stool transplanted mice. This indicates that the gut microbiome plays a part in ICP development via the FGF15-FXR axis and does not merely result after the hypercholanemia (2).

Despite remaining gaps in mechanistic understanding, the clinical implications are considerable. The gut microbiome may serve as a diagnostic biomarker, a tool for risk assessment, and a therapeutic target in ICP (2, 26). Given the maternal and fetal risks associated with the disease, microbiome-based strategies could complement current management approaches and potentially improve perinatal outcomes.

### 5.1 Limitations

Most of the included studies focused on the changes in the abundance of the gut microbiome, while only a few investigated its role in pathogenesis (1). As a result, there is great knowledge regarding changes in the gut microbiome across all taxonomical levels, but there are few explanations for the mechanisms by which they occur. Moreover, the laboratory testing methods differed across the studies and precluded the possibility of a statistical analysis. This points out two crucial limitations: lack of standardization in methods and geographic/ethnic bias, both of which affect generalizability.

The fact that the majority of ICP microbiome studies are conducted at Chinese research institutions raises fundamental questions about their generalizability, as both dietary habits and genetic factors significantly influence gut microbiome composition and ICP pathogenesis. A high-fat diet has been shown to increase total and secondary bile acids in the gut while simultaneously promoting the growth of bacteria with bile salt hydrolase activity (88). A high-fiber diet, on the other hand, supports the production of short-chain fatty acids, improves microbial diversity, and reduces potentially pathogenic bacteria, while positively influencing bile acid metabolism. In addition, polyphenols and prebiotics can modulate bile acid synthesis via the FXR/FGF15 axis as well as through hepatic metabolic pathways (89). However, these effects are strongly influenced by gene–diet interactions, such that both genetic variants (e.g., at the LCT locus) and different dietary habits lead to population-specific microbiome profiles (90). As a result, both the “baseline” microbiota and the observed dysbioses may differ, which complicates the generalizability of study results and therapeutic approaches, particularly in the context of ICP.

This literature review included 21 articles, which varied widely in terms of the information relevant to this topic. In some articles, ICP and its relationship to the gut microbiome were only a minor topic, but they were included due to the limited number of suitable articles. However, these did not significantly influence the conclusions of this study. As previously mentioned, most papers stem from Chinese research laboratories with high research output, only few stem from western research facilities. Also, the laboratory methods varied significantly, making a statistical analysis of the results impossible, which would have been highly valuable to the research field.

### 5.2 Clinical implications

Current research on ICP-microbiome interactions remains in preliminary stages. Nonetheless, these findings demonstrate considerable promise. The gut microbiome may offer significant potential as a biomarker, disease modifier or pharmacological target. A potential approach could involve the screening for ICP. Screening for gut microbiome profiles that predispose individuals to ICP could help identify women at risk, allowing an introduction of targeted interventions in high-risk population prior to disease development. This systematic literature review confirms that the current state of research is still far from standard of care as no reliable biomarkers for ICP were identified and no established treatment options for ICP are available. Notably, the implementation of screening programs depends on the ICP prevalence in the respective area.

The pathogenesis of ICP, particularly the interaction between the gut microbiome and BA, require further investigation to detect relevant biomarkers for the diagnosis and therapy of ICP (1). Screening methods could focus on the detection of rare, pathognomonic bacterial species, the serum metabolome in stool samples or the detection of genetic mutations that predispose individuals to ICP. Also, one would need to weigh the cost–benefit ratio of these methods and identify which target group is screened for ICP. Prior to implementation of a screening program, it is essential to establish adequate ICP therapy and develop a reliable screening method. Considering the serious consequences of ICP, a screening could certainly be warranted.

Multiple studies investigated new therapeutic approaches for ICP, as currently no curative therapy with sufficient evidence to improve maternal symptoms and fetal outcomes exists. The FGF19-FXR pathway could represent a promising drug target, as its upregulation has shown to lower the excessive BA synthesis and increase the mRNA levels of important export transporters for BAs (2). Some of the included studies researched FXR agonists, such as CDCA, as therapy options (79).

The mechanism and role of probiotics in ICP therapy is being investigated as well and could prove to be a cornerstone of ICP therapy or prevention in predisposed populations. Additionally, the microbial dysbiosis, observed in ICP patients, appears to be linked to the characteristically altered BA metabolism of the condition. Therefore, potential therapeutic targets, such as restoring SCFA-producing bacteria or modulating BA-related microbial pathways, could alleviate symptoms or reduce ICP risk in predisposed individuals (1). The species *B. fragilis* and its impact on the BA metabolism makes it a potential target for ICP treatment, a modulation of gut microbiota might reduce disease severity (2). Given the importance of the gut microbiome and its metabolites in ICP aetiopathogenesis continues to be recognized as central, stool transplants might be considered a viable therapeutic option for severe cases of ICP.

### 5.3 Future research

As mentioned above, our knowledge of how this gut microbiome shift in ICP pregnancy is limited. On the other hand, we know how the gut microbiome compositions and taxonomical shifts occur in ICP. The detailed steps should be further elucidated in order to understand the disease mechanism (for example using omics approaches) and find leads on where therapeutic interventions on the gut microbiome could be applied. Studies using germ-free mouse models can provide valuable insights into the specific role of the gut microbiome in ICP pathogenesis and help identify specific pathognomonic bacterial genera (1). Finally, mechanistic studies should expand the knowledge beyond taxonomy or functional studies and introduce the role of probiotics in clinical practice. The results could pave the way for developing therapies aimed at correcting gut microbiome dysbiosis in ICP. Since certain transporter mutations are associated with a higher ICP prevalence, exploring genetic diagnostic or therapeutic tools would also be of interest as well. Furthermore, the altered metabolism pathways in ICP could be associated with the corresponding increased or depleted bacterial genera, altering their metabolite profiles, and represent a potential target for therapy.

## 6 Conclusion

ICP is a pregnancy specific primary liver disease, which typically occurs in the late second to third trimester. This condition poses significant fetal risk, even lead to stillbirth. Previous research on ICP and its links to the gut microbiome has yielded promising results. The gut microbiome is constantly changing during pregnancy and influences the bile acid metabolism. The mechanism underlying these interactions are currently under investigation. In severe ICP, a distinct gut microbiome composition can be described. Most differences in abundance, compared to healthy pregnancies, can be observed in the phyla *Firmicutes* and *Proteobacteria* (46). In ICP, SCFA-producing genera are depleted (26). Also, the changes in BA profiles typical in ICP, influence the gut microbiome composition, particularly by favoring bile-tolerant species (15). Researchers conclude, dysbiosis plays a part in ICP pathophysiology (46).

Currently, there is no evidence-based therapy approach to ICP, which benefits the mother and fetus. There are several research projects aiming to target the condition. However, further investigation is needed and may contribute to establishment of standardized treatment regimen or the development of screening programs. The partaking of the gut microbiome in ICP pathogenesis implies its central role in future ICP treatment, as screening biomarker, pharmacological target or even as therapeutic agent.
